# Lactose Intolerance, Dairy Avoidance, and Treatment Options

**DOI:** 10.3390/nu10121994

**Published:** 2018-12-15

**Authors:** Andrew Szilagyi, Norma Ishayek

**Affiliations:** Department of Medicine, Division of Gastroenterology, Jewish General Hospital, McGill University School of Medicine, 3755 Cote St Catherine Rd, Room E110, Montreal, QC H3T 1E2, Canada; norma.ishayek2@mcgill.mail.ca

**Keywords:** lactose, intolerance, maldigestion, effects on diseases, specific therapy

## Abstract

Lactose intolerance refers to symptoms related to the consumption of lactose-containing dairy foods, which are the most common source for this disaccharide. While four causes are described, the most common is the genetically-determined adult onset lactose maldigestion due to loss of intestinal lactase governed by control of the gene by a 14,000 kb promoter region on chromosome 2. Gastrointestinal symptoms from lactose have expanded to include systemic effects and have also been confounded by other food intolerances or functional gastrointestinal disorders. Partly because lactose maldigestion is often interpreted as lactose intolerance (symptoms), focus of therapy for these symptoms starts with lactose restriction. However, withholding of dairy foods completely is not appropriate due to a more favorable impact on health. Industrial efforts to substitute with plant-based products is not completely successful at this time. This narrative article reviews the complexities of the perception of lactose intolerance, its epidemiology, and pathogenesis. Treatments are discussed, including the inappropriateness of dairy avoidance. In conjunction, effects of dairy products on 19 common diseases are reviewed. Different methods of treatment, lactose-reduced products, plant-based dairy substitutes, adaptation, prebiotics, exogenous lactase, probiotics, and some other dietary interventions are further discussed.

## 1. Introduction

Symptoms allegedly related to consumption of lactose have captured the interest of both the scientific community and the public. When one uses the popular web search engine Google for lactose intolerance, more than 8 × 10^6^ sites appear in seconds. The discovery that lactose digestion capability is a genetic trait, which divides the world into two phenotypes, has introduced the concept of symptoms caused by foods and nutrients. From the beginning of the second half of the last century there has been a debate on the role played by lactose-containing foods as they relate to symptoms similar to those caused by any maldigested/malabsorbed carbohydrates. More recently other dairy food related nutrients, like some fats and casein proteins, have been recognized to contribute to digestive symptoms. In parallel to the processes by which foods cause symptoms, the recognition of the role of the intestinal microflora in multiple normal and pathological host interactions complicated the explanations of why people have symptoms after eating. Lactose remains unique among nutrients because its source is largely confined to mammalian milks and its metabolism is genetically modified. As such, on a clinical level, lactose serves as a primary model for food-induced symptoms. Nevertheless, its role may be widely overstated because it is difficult to distinguish from different causes of similar symptoms.

On a population level diseases traditionally related to western lifestyles have been related to increased rates associated with higher latitudes and lower sunshine exposure and, thus, lower vitamin D [1,2]. Distributions of LP (lactase persistent) and LNP (lactase non-persistent) populations may also have impact on many facets of human life. The simplest examples of this impact are the different quantities of dairy food that make up daily intake of LP and LNP peoples. It has often been documented that LNP persons consume less dairy than LP [3,4,5]. The reasons for this may be largely cultural, but adult LNP persons not accustomed to regular dairy intake may experience a host of gastrointestinal and systemic symptoms. These symptoms and the putative mechanisms of formation are discussed later.

Another difference between LP and LNP populations is that due to a reduction of intestinal lactase, consumption of lactose quantities which overwhelm residual lactase are handled by intestinal bacteria (bacteroides, clostridia, and others as well). These bacteria ferment lactose into gases, hydrogen carbon dioxide, and in those who contain an abundance of Archea; methane. In addition, short-chain fatty acids are produced (SCFA, which are four-carbon organic acids; acetate, propionate, butyrate, lactate, and formate). These metabolic products, and their rate of production, can contribute to symptoms of LI (lactose intolerance) [6].

Lactase distributions are also related to the geographic patterns of some diseases [3,7]. The explanations for associations are not forthcoming. Some effects may be due to increased dairy intake or diminished dairy intake. However, other associations, such as genetic, economic, and environmental factors, may play independent effects. Relationships between diseases and lactase distributions will likely change as population migrations shift.

This review will outline “classical” lactose intolerance and differentiate other nutrients which cause similar symptoms. The article will review treatment options which focus on lactose and discuthe nutritional value of dairy products versus that of plant-based substitutes.

## 2. Lactose Sources, Synthesis, and Metabolism

**Figure 1:**
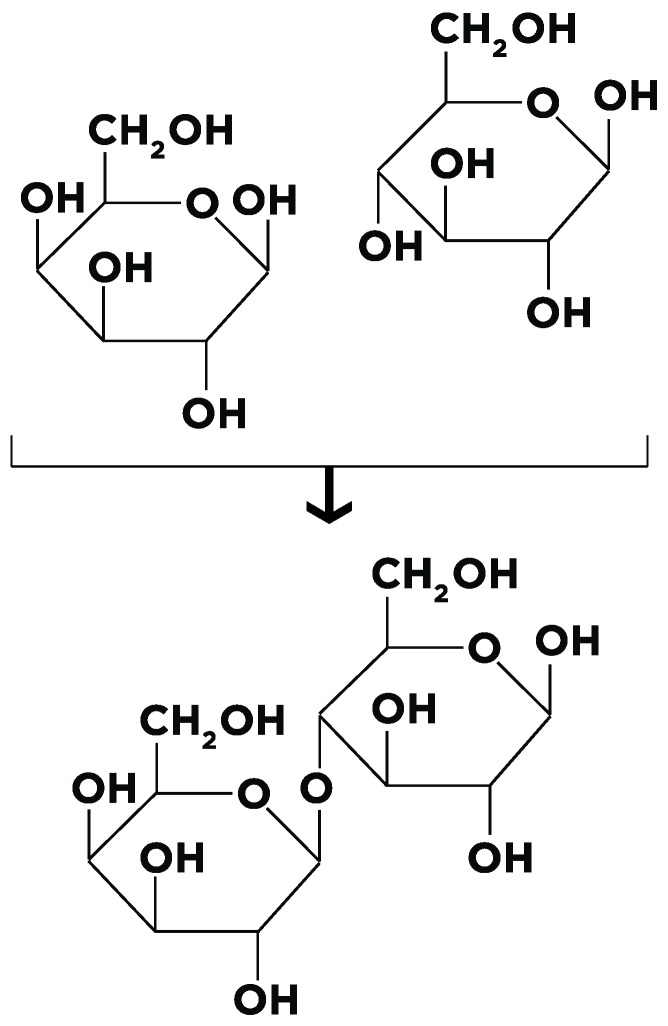
A molecule of the disaccharide *β*-d-lactose and the two molecules which make up lactose are shown. On the left is galactose, while on the right are two glucose molecules attached to each other by a 1-4 glycosidic bond.

Lactose is a disaccharide consisting of d-glucose and d-galactose. Biochemically, it contains two aldohexoses and is classified as O-*β*-d-galactopyranosyl-(1-4)-*β*-glucose (Figure 1).

Lactose represents the main carbohydrate of mammalian milk and very few other sources of this carbohydrate occur in nature [8,9]. Its detection in plants is debated [10]. Synthesis of lactose in the mammary gland depends on activated uridine di-phosphate galactose which is combined with glucose by the enzyme galactosyl transferase. The process of lactose synthesis is modulated by prolactin which increases post-partum and diminishes with weaning in conjunction with decreasing progesterone levels [8]. Human milk contains about 70 g/L (7%) of lactose which provides about 30–40% of calories to neonates [11]. In human milk about 5–8 g of galacto-oligosaccahrides provide important benefits to the neonate while increasing beneficial intestinal microbes [12]. In comparison, that of bovine milk contains about 46 g/L (4.6%) [13].

Lactose digestion and assimilation depends on the presence of the proximal intestinal brush border enzyme lactase phlorizin hydrolase (LPH). Two intraluminal enzymatic sites project into the lumen of the gut, which splits the disaccharide into glucose and galactose. These monosaccharides are then carried via the sodium glucose transporters across the intestinal brush border. Glucose is utilized mostly for energy; however, galactose is utilized by the neonate for multiple purposes. These include energy and structural molecules used in cell–cell communications, immune functions, epithelial stabilization, and neurological development. Metabolism of galactose requires detoxification via a unique mechanism: the four enzymes of the Leloir pathway. This pathway exists in most unicellular and multicellular organisms [14]. Alterations in Leloir pathway enzymes lead to rare genetic metabolic diseases classified as galactosemias [15]. 

The other major substrate of LPH situated on a separate site is the flavonoid phlorizin which is largely derived from the bark of the apple tree and its functions in mammals are not clear [16]. The site which digests phloirizin is also responsible for splitting glycosylated ceramides. Ceramides are functional lipids used by the host for cell communications similar to that outlined for galactose. In fact most ceramides are conjugated with galactose [16,17].

Weaning after breast feeding in all mammalian neonates may be triggered by diminishing lactose content in mammary glands of the mother. This event correlates with diminishing intestinal lactase levels in the infant [8]. The complex physiology of breast feeding and weaning are reviewed by Lawrence and Lawrence [18]. Intestinal LPH levels diminish to about 10% of neonatal levels of most mammals. In small animals like the mouse and rat, refeeding lactose containing food can lead to increasing intestinal LPH, that is, the enzyme is inducible. In humans, however, lactase is not inducible [19].

In the second half of the 20th century researchers discovered that LPH is genetically controlled and the ability to retain lactase into adulthood is a dominant trait [20]. The gene for LPH is located on chromosome 2q21 [21] and is controlled by a promoter region approximately 14,000 bp upstream of the translation initiation codon of the lactase gene (LCT), 

The first polymorphism in the promoter region upstream from the lactase gene was described by Ennatah et al. and was found in most Europeans who carry the −13910C > T (rs4988235) variant (TT) [22]. A single T-allele change at position 13910 in intron 13 of the minichromosome maintenance complex component 6 (*MCM6*) gene controls LPH [20,22]. Since then, four other genetic variants have been identified to have co-emerged across different geographical regions [23,24,25]. The transcriptional control of the lactase gene prevents its downregulation. The exact factors responsible for the lactase enzyme regulation have not been fully worked out but involve epigenetic methylation of DNA [26,27]. While the C variant DNA is methylated and transcription is inhibited, the T variant is less affected. The upregulation of the T variant involves binding to several transcription factors (Oct-1,CDX, HNF1α, GATA 4/6) which bind the TATA-Box (a DNA sequence indicating to other molecules where transcription begins) in the lactase promoter region. The -13910 T enhancer binds closely with the Oct-1 transcription factor and also with HNF1α [28,29,30]. Recently, the intestinal peroxisome proliferator-activated receptor gamma (PPAR_γ_) transcription factor, which participates in glucose and lipid metabolism, has also been shown to upregulate lactase both in an in vivo rodent model and in the human intestinal cell line Caco-2 [31]. 

**Figure 2:**
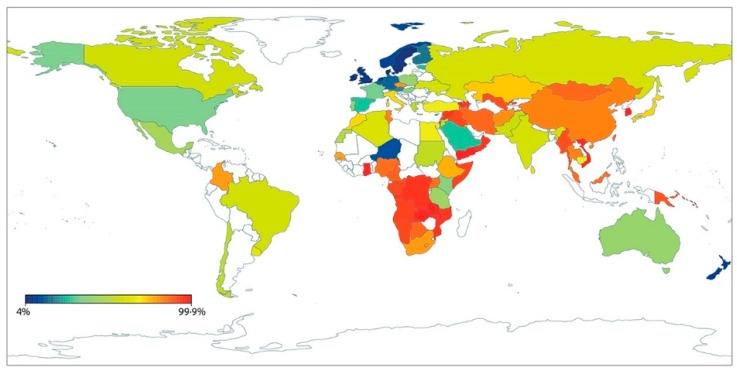
Representation of the geographic distributions of lactase non persistence frequencies of populations in the world.

Epidemiological studies established that while about 2/3–3/4 of the human population follow other mammalian example and reduce LPH to 10% of neonatal levels, about 1/4–1/3 retain the ability to continue to digest lactose into adulthood. Persons with this dominant ability are lactase persistent (LP) while those with the recessive form are lactase non-persistent (LNP). There are distinct geographic distributions of LP and LNP populations. While different explanations were considered to account for such distributions the current prevailing one is the gene-culture-coevolution hypothesis [32]. This suggests that populations with a background of domesticating and consuming mammalian milks migrated to different geographic regions of the world. Increased migrations subsequently, during and after the discovery of new worlds, helped shape world distributions of LP/LNP populations. This migratory alteration of distributions continues today as can be deduced from a recent publication of LP/LNP distributions from some 86 countries [33]. The map (Figure 2) is reproduced from a publication by Storhaug et al. Reprinted from The Lancet Gastroenterol Hepatol. 2017, Vol 2(10), [30] with permission from Elsevier.

## 3. Causes of Lactose Maldigestion

Four principal situations exist where lactose may be maldigested. The first is rare and is due to recessive genetic mutations in the intestinal lactase enzyme. Many, but not all [34,35], cases have been reported from Finland [36,37]. In these cases neonates are unable to digest lactose and clinical symptoms rapidly develop with the beginning of breast feeding. Often there is severe diarrhea, acidosis, and hypercalcemia [38]. The therapy is to avoid all forms of milk. 

In premature neonates intestinal lactase may not be fully developed and this situation constitutes the second form of lactose maldigestion. There is some controversy as to when intestinal lactase matures in term infants. Some authors suggest that within five days of birth the enzyme is fully available [39]. Others find that, in some neonates, lactose spills into the large intestine contributing to colonic microfloral changes which aid in neonatal nutrition [40].

The most common form of lactose maldigestion is adult onset hypolactasia, which is genetically controlled as described. The majority of the discussion of lactose maldigestion relates to this form. The distribution of LP/LNP phenotypes follow ancestral distributions in the old world. In the new world LP/LNP distributions depend on the admixture of original native populations, founding populations spanning several centuries and more recently the migration of populations from developing nations to the new world. In largely LNP populations the phenotype manifests at a relatively young age, 2–5 years [41,42]. However, in mixed LP/LNP populations the LNP phenotype can manifest in the teenage years [43].

The fourth form of lactose maldigestion relates to diseases causing loss or injury to the small bowel. In this situation intestinal villi are lost and with it there is a reduction in intestinal lactase leading to lactose spill into the lower intestine. Diseases which affect the small bowel mucosa include gluten sensitive enteropathy, lymphoma infections like giardia, small bowel bacterial overgrowth, and others. These diseases can, of course, affect either LP or LNP persons and the severity of symptoms may depend on the extent of mucosal involvement and underlying genetic predisposition. Acquisition of such diseases can unmask lactose maldigestion in LNP persons who were consuming dairy products regularly and the genetic status then lingers revealing adult hypolactasia. These causes are outlined in Table 1.

## 4. Lactose Intolerance

The classical term lactose intolerance (LI) has been applied to the development of gastrointestinal symptoms (of gas, bloat, abdominal cramps, and pain sometimes associated with mushy to watery diarrhea and, on occasion, with nausea and vomiting) after ingesting large amounts of lactose-containing food. Adult onset lactose maldigestion (LM) has often been interchanged with LI. However, the term can be confusing because LI does not always follow LM. Additionally, self-belief of LI influences reaction to dairy foods without proof of LM. Moreover, the term LI has frequently been noted with lactose load challenge tests and the presence of these symptoms in LNP people and has been classified as intolerance. The result may not reflect lactose related symptoms in daily life. These terms are shown in Table 2.

### 4.1. Diagnosis of Lactose Maldigestion

There have been different tests designed to evaluate the person’s response to lactose loads but most are now delegated to 2–3 tests. The most common one in use is the lactose breath hydrogen test. After a lactose load at doses ranging from 20 to 50 g exhaled breath hydrogen and methane are measured with an electrochemical sensor or a gas chromatograph. A value above 20 parts per million denote fermentation by intestinal bacteria of carbohydrate that has not been absorbed by the host [45]. This test may detect genetic heterozygote persons by comparing different areas under the curve [46]. Additionally, by combining test results with methane production, a more accurate detection of lactose maldigestion may be obtained [47]. The second most common test is a blood test where a failure to raise blood glucose above 1.1–1.4 mmol/L suggests lactose maldigestion. Here the host’s intestinal enzyme levels are directly tested as opposed to the breath test where malabsorbed lactose is used to test the absence of the enzyme indirectly. The two tests have discrepancies for different reasons [48,49] but individual results do correlate reasonably well with genetic tests [49]. Other tests also exist [50] and some are used more commonly in children than in adults [51]. Intestinal biopsies which measure intestinal enzymes are too invasive for general population evaluations. A low stool pH test (denotes acidification) can be used for children as an indirect measure for carbohydrate malabsorption.

### 4.2. Pathophysiology of Symptoms Related to Lactose

Symptoms of LI are considered to be caused by intraluminal osmotic forces which depend on the rapidity and the quantity of undigested disaccharide entering the small and large bowel. Rapid bacterial fermentation of lactose to gases and SCFA contribute to symptoms [6]. This scenario occurs most often in LNP persons with infrequent dairy consumption or recent development of secondary lactase insufficiency. Symptoms which can be directly connected with consuming lactose in LNP persons constitute classical LI. Symptoms from LI are usually detected and classified during lactose challenge tests. From these it has been learned that several variables can modify these symptoms.

In this context, the dose of lactose consumed plays an important role. A number of studies bear on the interaction between intestinal lactase and a single-dose ingestion of lactose. In a landmark study by Bond and Levitt it was noted that even LP persons with full complement of intestinal lactase could spill about 8% of an ingested dose [52]. However, when one evaluates lactose tolerance in LNP persons the impact of residual LPH is more acute. Several studies have evaluated the minimal dose of lactose tolerated without symptoms [53,54,55,56,57]. In general, the conclusions from these studies was that most lactose maldigesters are able to handle the amount of lactose in a cup of milk (12.5 g/250 mL) and have minimal to no symptoms.

A frequent concern is the relevance of lactose incorporated as a filler in medications [58]. The amount of lactose in a single ingestion of medicines is far below the 10–15 g which is tolerated by lactase-persistent persons. In a controlled trial using breath test analyses, the amount of lactose present in medications (average 400 mg) did not lead to a positive rise in breath hydrogen suggesting absorption by the host [59]. 

Consuming lactose with meals, especially fat (in milk or otherwise), slows gastric emptying, reducing the quantity of lactose exposure to the small intestine per unit time [60,61]. On the other hand foods like coffee or hot peppers may increase intestinal transit delivering lactose to the lower intestine and increasing symptoms. Lactose in fermented dairy products contain quantitatively less lactose volume for volume. Products like yogurt contain lactic acid bacteria which reduce the lactose content through bacterial galactosidase metabolism. 

Intrinsic transport time of gastric emptying or intestinal transit can also alter the rate at which non absorbed lactose is fermented. The altered gastric emptying or intestinal transit (delay or increase) can be affected by physiological conditions like pregnancy [62], medications [63], or diseases (diabetes, thyroid disorders, collagen vascular diseases) [64].

Therefore, one can perceive LI as an interaction between level of lactase and the dose of single ingestion of lactose. In this spectral scenario all causes are included depending on genetics of the person, diseases that affect the small bowel, or the vehicle by which lactose is ingested.

### 4.3. The Impact of Knowledge about Lactose Intolerance on Dairy Avoidance

The relationship between gastrointestinal symptoms and lactose consumption has a somewhat historical basis. Such nonspecific symptoms have been recognized for a long time and have also been diagnosed under the term Irritable Bowel Syndrome (IBS). This condition, with other functional gastrointestinal symptoms, have generally been classified based on symptomatic and timeline criteria. These have gone through a number of modifications through selective meetings under the Rome Criteria [65]. IBS represents one form of these gastrointestinal disorders. The gastrointestinal symptoms of LI can be very similar to those of IBS. In the second part of the 20th century when lactase and its relationship with lactose was described, the cause for IBS seemed to be discovered. Lactose in LNP persons caused bloating, gas, and even diarrhea without apparent pathological alterations in the gastrointestinal tract. Since LNP status could only be detected by specific tests, it was natural to consider LM to be the cause of IBS. This however is not the case.

When patients blame LI for symptoms an important question when evaluating lactose intolerance is whether patients’ association of gastrointestinal symptoms with ingestion of lactose is a reliable predictor of lactose maldigestion. Self-reported lactose intolerance refers to the belief that the person is likely to suffer gastrointestinal symptoms after consuming lactose. It does not include clinical tests for lactose digestion. In a meta-analysis of 21 studies comparing various doses of lactose with placebo in patients with gastrointestinal symptoms, different outcomes for abdominal symptoms reported minimal to zero differences between the groups [66].

Self-reported lactose intolerance is an unreliable diagnostic tool for lactose maldigestion. In a systematic review of 26 studies, evaluating the reliability of gastrointestinal symptoms to predict malabsorption, Jellema et al. found a highly variable outcome. When breath hydrogen outcomes were evaluated only a non-Caucasian ethnic origin was predictive of positive tests. Both lactose absorbers and maldigesters reported symptoms during testing [67].

During the last decade another dimension was attributed to lactose induced symptoms. Mathews et al. described a patient who suffered systemic symptoms after consuming lactose and completely recovered when lactose was withdrawn from the diet [68]. Later, these symptoms were renamed as lactose sensitivity and were widely described in patients with inflammatory bowel disease [69,70]. Others have also attributed mood changes which accompanied carbohydrate ingestion, especially lactose intolerance [71].

In accordance with SRLI a placebo effect and a nocebo effect (the belief that what is consumed in a blinded exposure is lactose, leads to the experience of typical lactose--induced symptoms) have been described [72]. In the first instance regular consumption of lactose reduces classical symptoms. Although colonic adaptation likely explains some improvement, a placebo effect with diminishing perception of severity of symptoms have been hypothesized [73]. In the second instance Vernia et al. reported on the finding of typical LI symptoms in patients with negative breath tests where false negative results was eliminated [74].

These analyses suggest that the population-reported notion of lactose intolerance as a cause of multiple symptoms does not predict maldigestion of lactose. It is entirely possible then, that the subjective complaints of symptoms merges with the concept of functional gastrointestinal disorders (FIGD). These disorders consist of a set of symptoms involved with different locations in the gastrointestinal, gynecological, and other systems [75,76]. All are diagnosed based on specific sets of symptoms and associated with time dimensions, Most relevant of these functional disorders the irritable bowel syndrome (IBS) has the closest semblance and may mimic lactose intolerance. The cause(s) of IBS, to date, is not based on defined pathology and is thought to be related to interactions between the brain and gut microbiome, as well as gut and central neurologic communications [77,78]. The rate of SRLI in predominant LP populations is about 15–16% [79,80] and, interestingly, the rate of SRLI is reported to be similar in IBS patients from LNP dominant populations [81].

### 4.4. Merging of Lactose Intolerance with Symptoms of Other Disorders

An important question that still remains regarding lactose, concerns the role of this disaccharide in causing symptoms in other disorders. There is a wide discrepancy between public perception of LI and other causes of similar symptoms. While lactose is the perceived focus, there may be relationships with other nutrients in dairy foods, in general.

In the last three decades the potential involvement of LI-like symptoms have been expanded. However, the causes include different etiologies. The first confounder of LI is IBS.

As stated, IBS is a collection of symptoms with a recurrent time variable as defined by the Rome criteria [65,75]. Classical IBS is divided into four types: IBS-Diarrhea, IBS-Constipation, IBS-Mixed, and IBS-Undifferentiated [77]. The associated types depend on the frequency of each feature more or less than 25% of the times. Since there is no specific defining pathology at this time treatments are symptom-based with variable outcomes of efficacy. The current explanations of symptoms is a bidirectional interaction between intestinal microflora communicating with the enteric and subsequent central neurologic system [78,82]. This paradigm is also reminiscent of the pathogenesis of LI proposed by He et al. [6]. The hypothetical question of whether IBS was specifically caused by LI emanated early on after the discovery of the biochemistry of lactase and its genetic nature. However, the distinction between IBS and classical LI was established with multiple studies [83,84]. Epidemiologically, the prevalence of lactose maldigestion follows relatively distinct geographic patterns, while that of the prevalence of IBS is more independent of LM [85,86]. Perhaps because of increased visceral sensitivity postulated to be part of functional gastrointestinal disorders LNP persons may, nevertheless, be more sensitive to lactose challenges than LP persons [87].

Additionally, in the last three decades, other nutrients gained interest in the possibility of causing LI-type symptoms with consumption of dairy products. Mishkin drew attention to the possibility that certain fats present in some dairy products were more likely to cause symptoms, for example, when consuming ice cream [88]. In a study from New Zealand evaluation of the effect of dairy food intake in patients with inflammatory bowel disease Nolan-Clark et al. noted that symptoms correlated better with fats than lactose [89].

More recently the possible impact of dairy protein, casein, was proposed to induce LI type symptoms. Genetic differences in cows lead to either A1 casein (more European types) or A2 (more associated with Asian cows). The A1 casein is hypothesized to interact with µ receptors in the gastrointestinal tract and induce motility which can cause abdominal symptoms [90,91]. However further work is needed on these findings to establish the role of casein in different populations. 

Finally, allergy to cow’s milk protein (whey and casein) is largely limited to children and is very different from lactose intolerance [92]. The allergens are thought to be to *α*s1-, *α*s2-, *β*-, and *κ*-casein and *α*- and *β*-lactoglobulin proteins [93]. Allergic reactions could be IgE mediates, but the majority of cases are not related. Symptoms could include respiratory, skin and gastrointestinal sources [91]. The latter sometimes resembling inflammatory bowel disease with diarrhea and bleeding [94]. Occasionally cow’s milk protein allergy can occur in adults as well, but most present without gastrointestinal symptoms [95]. The majority of gastrointestinal diseases and symptoms in adults are not milk allergy dependent [96]. 

In view of perceived symptoms from dairy consumption, the notion of eliminating dairy from diet may be entertained. Since dairy is regarded as highly nutritious, a review of the effects on various conditions is provided in the following section.

## 5. Consequences of Dairy Product Consumption

One of the simplest methods is to eliminate dairy foods from diet. However, the conclusion of an NIH single-topic conference on lactose intolerance concluded that the main health impact of this condition is the withholding of milk and dairy products [97]. This section will review relations between dairy consumption and a number of conditions. With the exception of bone, where dairy supplies the largest amount of calcium, relations with other diseases are often modest, associated with reduced risk, or are neutral, and occasionally increase risk, but are not considered to be causative.

### 5.1. Bone Formation, Maintenance, and Bone Disease

One of the most important relationships is between dairy intake and bone dynamics which is constantly remodeled throughout life (development, maturation, maintenance, and osteoporosis). Bone turnover is increased with aging and in a number of pathological states, such as osteoporosis. The increased bone turnover leads to deterioration of bone microarchitecture which then, independently of bone mineral density, can lead to fractures [98]. Bone modeling is the primary organ that relies on appropriate calcium intake. The requirement for calcium in bone health has been established. Calcium, vitamin D, and protein are needed for the maintenance of bone mass, architecture, as well as for modeling throughout life [99]. In addition, vitamin A, potassium, zinc, and magnesium in dairy products are also important nutrients in bone formation. These requirements are essential during adolescent bone formation and may be less important in adults.

In a review of 52 randomized and 89 observational studies Heaney concluded that intake of these nutrients for proper bone status is supported by the majority of reviewed studies [100]. These reviews concluded that dairy foods are an excellent source for the nutritional requirement for healthy bone status and that it is difficult to attain recommendations to consume enough calcium without use of dairy. In the United States calcium intake is frequently less than the recommended amounts in adolescents (especially females-1300–1500 mg/day, 37.5–60 mmol/day or post-menopausal females (1500 mg, 60 mmol/day) [95]. The concept of SRLI further contributes to reduced calcium intake. A study of self-imposed dairy restriction in young girls (ages 10–13) led to an approximate 210 mg calcium intake deficit compared with girls without SRLI [101]. About half of these young girls were lactose digesters. 

Since dairy supplies calcium and other important nutrients for bone health, the reduction or elimination of dietary dairy products raises a question of whether lactase non-persistence status (known for reduced dairy consumption) may predispose to osteoporosis. The basis for the query is that LNP populations consume less dairy than LP populations [3,4,5,102]. In turn, the reason for this could be an increased development of gastrointestinal symptoms from the different way lactose is handled by LNP persons [6,103]. Other reasons for low dairy consumption likely relate to diet influenced by cultural differences [104]. A recent study from Italy found that in adult-type hypolactasia (LNP) even the consumption of lactose-free milk led to lower than recommended calcium intake [105]. Indeed, an evaluation of global dietary calcium intake, based on 74 countries, found large differences in various regions of the world. Asians (high LNP) generally consume <500 mg/day. In Africa and South America (approximately mid-LNP) consumption is 400–700 mg/day, and in Northern Europe (low LNP) consumption did achieve >1000 mg intake/day [106].

The ongoing controversy is whether LNP and low dairy consumption lead to higher rates of osteoporosis and fractures. There are reports that LNP persons consume less milk calcium which leads to reduced bone density and increased fractures [107,108]. However, others noted that the presence of lactose maldigestion, per se, does not lead to reduced bone density [109], unless associated with reduced calcium intake [110] and severe intolerance symptoms [111]. 

On epidemiological grounds there is little information on national prevalence rates of osteoporosis. A review by Wade et al. found the highest rate for women in Japan (>73% LNP rate) and the lowest rates in the United Kingdom (8% LNP). For men the distinctions were less clear [112]. In another report Hernlund et al., from the European Union, found that Germany (16% LNP) had the highest prevalence of osteoporosis and Italy (72% LNP) had the second highest. Moreover, a compilation of fractures showed the highest pattern in Denmark, with Tunisia the lowest [113]. These two reports lead to two observations. There may be a weak correlation between national LNP rates [33] and osteoporosis rates, however, methods of osteoporosis definitions vary. Second there is a discord between osteoporosis rates and national fracture prevalence. The reasons for these discrepancies are not clear. Nevertheless, generally, the relationship between dairy intake and bone health is supported but is more substantial in children and adolescents than adults.

### 5.2. Cardiometabolic Syndromes

The potential benefits of dairy foods against cardiometabolic syndromes (these syndromes collectively refer to dyslipidemia (met S, high cholesterol or triglycerides both of which can promote atherosclerosis or, in the case of triglycerides, also fatty liver), hypertension (HTN), cardiovascular (CVD) and coronary artery disease (CAD), stroke, and type 2 diabetes (T2D), as well as obesity, remain controversial. Part of the confounding effects may have been related to attempts to link individual nutrients to specific health benefits. There are recent suggestion that the effects of dairy matrix rather than individual nutrients may be more important [114,115]. In addition, the recognition that LP and LNP persons may handle lactose somewhat differently and that this is genetically mediated may impose different parameters depending on genotype/phenotype.

A recent large systematic analysis of cardiovascular-related outcomes with different dairy products have been published by Drouin-Chartier et al. In this review different dairy products have been separately assessed. Studies including total dairy, low or high fat dairy, milk, cheese, yogurt, and fermented dairy were individually determined with each disease. These components were evaluated with the diseases listed above (except obesity). Evaluating CVD, the various forms of dairy had a neutral effect. Similarly, the relationships between CAD and different forms of dairy were deemed neutral. Interestingly in the case of strokes, total dairy, low fat dairy cheese, and fermented dairy showed a modest inverse relationship while regular high fat dairy yogurt and milk showed no association [116]. Analyses with metabolic syndrome were based on limited studies, but total dairy and milk consumption did vary inversely. Other products could not be appropriately assessed due to very limited available information.

High blood pressure is associated with cardiovascular disease and its complications. Despite advances in medical therapy more than half of patients are not adequately controlled [117] making lifestyle and diet important components of therapy. Among these dairy products have been considered important in maintaining blood pressure. A systematic review and meta-analysis of calcium intake found a modest inverse relationship with hypertension [118]. In addition, several other observational systematic reviews and meta-analyses [119,120,121], as well as controlled trials of milk [122,123], showed benefit. The latter controlled trials suggested that milk peptides could be the active components responsible for the modest benefits. The analysis of total dairy, low-fat dairy, and milk was also found by Drouin-Charier et al. to be inversely associated with HTN [116].

The main conclusions from a review of interactions between aspects of the metabolic diseases and dairy consumption is that regardless of fat content or form of dairy there is no detrimental impact. There is high-quality evidence that low-fat dairy and yogurt reduces risk for T2D. There is moderate-quality evidence that risk for metabolic syndrome is reduced by total dairy and milk, while total dairy and cheese reduce risk for T2D. In the case of CVD and CAD most studies on dairy are neutral. Moderate-quality evidence favors beneficial interactions between total dairy, low-fat dairy cheese, and fermented dairy and stroke.

When genetic effects are taken into consideration by using Mendelian randomization methods (to reduce confounding and possible reverse causation [124]), different outcomes are obtained. Hartwig et al. suggested that while the dominant genetic trait of the European C/T = 13,910 polymorphism (rs4988235) is associated with an increased BMI it was not associated with hypertension. They showed that there was a different outcome between observational studies and using Mendelian randomization. In the latter analysis any advantage dairy products had were negated [125]. A similar outcome failed to show a beneficial effect toward blood pressure control with dairy foods by Ding et al. [126]. However, a Mendelian randomization study to evaluate cardiovascular risks did not find increased risks with milk consumption [127] similar to observational studies.

Regarding type 2 diabetes, in the review of Drouin-Chartier et al. high-quality evidence supports an inverse relationship in risk between low fat dairy and yogurt intakes [116]. Moderate evidence supports an inverse relationship between T2D and total dairy and cheese intakes. An earlier systematic review also suggested that some studies of dairy interventions favored an increase in insulin sensitivity [128] and saturated fats derived from dairy were not associated with increased risk for T2D [129]. The evaluation of T2D in relation with dairy intake by using Mendelian randomization, however, failed to show any impact [130].

The association of dairy with obesity has also run a contradictory course. Systematic reviews and meta-analyses of the effects of dairy on different aspects of body weight tended to support a protective effect. However more recent analyses of randomized controlled trials showed either neutral effects or a benefit only in the short term with energy restriction [131]. When the genetics of lactase are included the beneficial effects were largely eliminated [132]. The last study by the Mendelian Randomization of Dairy Consumption Working Group concluded that dairy products promote obesity. There is a possible counter argument to this conclusion in that the TT lactase (C/T-13910) genotype may be associated with increased BMI and possibly introduce horizontal bias [124,131]. The conclusions need to be further evaluated, possibly in addition to other polymorphisms which regulate lactase.

### 5.3. Colorectal Cancer and Inflammatory Bowel Diseases (IBD)

Epidemiological studies evaluating the correlation between colorectal cancer (adenocarcinomas arising from polyps which develop in the colon or rectum) or IBD (consisting of idiopathic ulcerative colitis and Crohn’s disease involving different parts of the entire intestine) and lactase distributions appear to be inversely related with LNP [3]. As such, countries with high dairy consumption appear to have higher rates of these bowel diseases, with colorectal cancer and ulcerative colitis showing a statistically significant relationship. These calculations were centered around the year 2000, and since then rates have increased even more in developing countries with progressive industrialization. However, despite the geographic observations colon cancer rates actually were consistently shown to be inversely associated with dairy intake in observational studies [133,134]. Although no formal Mendelian randomization studies exist evaluating dairy products and colorectal cancer a geographic regional evaluation of observational studies from three regions of the world based on predominant phenotypes of lactase distributions in the world showed that dairy modestly protects in both high LP and high LNP regions [135].

Originally calcium was considered to be the primary anti-carcinogen and the dose of calcium was considered to be >1200 mg/day for anti-neoplastic effects [136,137]. Indeed, controlled trials of supplemental calcium supported this relationship [138,139,140]. However, the expected dose of calcium intake from dairy was not achieved in high LNP countries [106]. Furthermore, recent studies seem to negate the interventional effects of calcium and vitamin D on precancerous polyp growth [141] and even suggest that the combination may enhance the growth of another histological precancerous polyp: the serrated adenoma [142].

More recently, other nutrients than calcium and vitamin D, in dairy products, have also been attributed to possess antineoplastic properties (milk fat globule [138,143], medium chain triglycerides [139,144], conjugated linoleic acid [140,145], whey protein [141,146], and lactose [135].

In the case of IBD a similar (ecological fallacy type) relationship may exist between dairy consumption and geographic risk [3]. Three studies to date have suggested that pre-disease intake of dairy may reduce risk of Crohn’s disease [147,148,149] and possibly idiopathic ulcerative colitis [149]. In the largest study to date while there was no dose effect of total dairy or individual dairy products there was a statistically significant protective effect of milk consumption compared to non-consumers (OR, 0.30 with confidence intervals of 0.13–0.65). There was also a trend for ulcerative colitis [149]. Certainly, more studies are needed in this area to validate these findings and the possible mechanisms.

### 5.4. Other Cancers

A number of other cancers have an inverse epidemiological relationship with LP/LNP global distributions [3]. The crude implication is that such cancers are more common in regions where more dairy is consumed. However, the relationship with dairy consumption is not obvious and studies evaluating effect at population level needs to be examined.

The epidemiological relationship of ovarian cancer with dairy consumption was not statistically significant [3]. Cramer initially noted the inverse relationship between national rates of ovarian cancer and increasing LNP rates. He suggested that abnormal metabolism of galactose could lead to neoplastic changes in the ovarian epithelium such as that observed in severe cases of Type 1 galactosemia [150,151], but this was not confirmed in normal women [152]. Nevertheless, the observation of a potential connection between dairy consumption and ovarian neoplasms remains a contested issue. Some studies did find an association between dairy consumption including lactose [153,154] but not calcium consumption. Prospective cohort, but not case control studies, in a meta-analysis also confirmed a dose effect of total dairy, milk, and lactose on ovarian cancer [155]. Others found no association between dairy or calcium consumption but a higher risk with increased lactose intake [156]. Still three other studies failed to find any association between epithelial ovarian cancer (90% of all ovarian cancers) and lactose intake [157,158,159]. The most recent meta-analysis of 15 epidemiological studies found that calcium intake through diet (dairy or non-dairy) had a modest protective effect against epithelial ovarian cancer [160]. Diet plus supplement, however, failed to reach statistical significance. Eleven of the 15 studies emanated from the United States.

The epidemiological relationship of breast cancer to dairy intake was not statistically significant [3]. Nevertheless, population level evaluations mostly support an inverse effect with reduced risk. The protective anticarcinogenic effect of dairy is hypothesized to be through calcium, vitamin D, butyrate, lactoferrin, and conjugated linoleic acid [161,162]. The largest meta-analysis by Zang, which included five Asian studies among 27 evaluated, found that low fat dairy and yogurt significantly albeit modestly reduced breast cancer risk. The protective effect was noted both in American and Asian studies [163]. An earlier meta- analysis of 18 studies found an inverse and dose effect of total dairy, but not milk. Additionally, the relationship was more pronounced with low fat than high fat dairy [164].

There was also a statistically significant positive epidemiological relationship with LNP national status and stomach cancer, which superficially suggested a possible protective effect of dairy consumption. However, a largest meta-analysis of the relationship with dairy consumption, using 26 studies, showed a neutral non-promotional effect [165]. A subsequent meta-analysis suggested that total dairy intake was protective against this cancer in Europe and the United States, but not Asia [166]

In a similar manner the effects of dairy products on risk for lung cancer was found to be non-significant in a meta-analysis [167].

Two cancers which appear to be related to increased dairy intake are prostate and testicular neoplasms. In the case of the former a statistically significant epidemiological relationship was noted with increasing national dairy consumption [3]. Additionally, several meta-analyses support an enhancing effect of dairy foods on prostatic neoplasms [168,169]. Since LP populations are at an increased risk for the cancer, the possible direct role of the North European lactase genotype (C/T-13910) polymorphism was evaluated. However, in the study by Travis et al. the TT dominant genotype was associated with higher dairy intake, but not with higher risk specifically of prostate cancer. The authors concluded that higher dairy intake was the likely pathogenic influence [170]. Most recently the putative pathogenic mechanism of dairy was evaluated through a systematic review and meta-analysis of the effects on various insulin growth factors (IGF). This large review of 172 qualifying studies concluded that the impact is likely through elevation of IGF-1 with dairy intake [171]. Insulin-like growth factors are implicated in various disease states, including neoplasia [172].

The epidemiology of testicular cancer suggests a place and time increase. Northern and more recently also Western Europe has the highest rates [173,174]. Additionally, cancer rates are generally the highest in industrialized nations, except China, where it is stable [174]. Among several risk factors dairy consumption has been reported to increase risks [175,176]. However, a study by McGlynn et al. evaluated nearly 1700 cases with germ cell tumors and controls and reported that when several other risk factors are evaluated height was the single variable of significance and dairy intake or body size were not [177]. 

Total dairy products and milk, but not yogurt, were found to pose an almost two-fold risk for non-Hodgkin’s lymphoma. The authors concluded that specific dairy products need to be evaluated for possible effects [178]. In the review by Thorning et al. there were inconsistent or neutral effects of dairy in cases of bladder or pancreatic cancer [179]. Table 3 outlines approximate effects of different dairy products and some nutrients on diseases reviewed in the literature.

To summarize, it seems that the relationship of dairy intake with diseases is a highly complex. Conclusions listed are also noted in the review by Thorning et al. [179]. The best established requirement is for bone formation during adolescence. However, intake of necessary nutrients may be obtainable from sources other than dairy as seen in regions with high LNP populations. Observational studies tend to support a neutral effect on cardiovascular disease with an inverse relationship between several dairy foods and stroke. Similarly, observational studies tend to support a modest benefit for hypertension and type 2 diabetes. Weight reduction and prevention of obesity may be facilitated by dairy foods in the short-term and in conjunction with energy restriction. However, further contradictory outcomes are noted when studies are evaluated by the method of genetic Mendelian randomization. Evaluation of hypertension, obesity and type 2 diabetes in these studies fail to support beneficial effects. The reasons for discrepancies are not absolutely clear. There is some suggestion that lactase persistence status may enhance body mass index independently of dairy intake. The possible role of other lactase polymorphisms have not yet been evaluated for possible independent effects. It is not clear whether detailed evaluations of different dairy matrix on target outcome also show null effects. 

Among the effects on neoplasms, dairy is modestly protective against colon cancer. The effects on other cancers are less well established. Of these, negative impact on prostate and perhaps testicular cancer although is supported but not proven. Further work is needed on disease relationships with dairy.

## 6. Treatment Options for Lactose Intolerance

One of the main concerns of leading health institutions such as the American national institute of health for individuals who are lactose intolerant is not getting enough essential nutrients due to complete avoidance of dairy foods. As stated calcium and vitamin D are examples of such nutrients essential for bone growth and maintenance in children and adults, respectively [180,181]. Dairy foods provide more calcium, protein, magnesium, potassium, zinc, and phosphorous per calorie than any other typical food found in the adult diet [182]. Availability and the relative low cost of dairy products makes their consumption more convenient. While lactose intolerance is a real entity, in response to excess lactose intake, the general perception of lactose intolerance is made up of a number of conditions which present with similar symptomology. As such, treatments for LI are fairly specific but additional therapies are needed if lactose reduction by itself is not adequate. The following sections outline these options.

### 6.1. Lactose-Free and Lactose-Reduced Products

The avoidance of all dairy products in patients with lactose intolerance is no longer recommended. Most people with lactose intolerance can tolerate up to 12–15 grams of lactose per day. Strategies to increase tolerance of lactose containing foods with the goal of improving nutrition adequacy, avoiding deficiencies and improving symptoms [182] are summarized in Table 4. People with lactose intolerance should be encouraged to restrict rather than avoid lactose with the goal of including some dairy foods in the diet and to benefit from associated nutrients and their higher bioavailability. Consumers should be educated by health care providers on the nutritional differences between dairy products and the non-dairy substitutes and should be guided on healthy choices. The food industry can also do its part by improving product labels, indicating lactose content and avoiding misleading claims. The government would be wise to introduce legislation that standardizes the definition of “no lactose” and “reduced lactose” and to make lactose content mandatory on nutrition labels. 

### 6.2. Non-Dairy Substitutes and Comparison of Inherent Nutrients with Dairy Products

Consumption of non-dairy substitutes has been on the rise and the food industry has responded by making these products more available on supermarket shelves. These products are primarily derived from plants, such as soy, rice, hemp, oat, coconut, almond, and other nuts. They can be fortified with one or more of the following: calcium, vitamins D, A, B12 and Riboflavin or none at all. Some manufacturers use the word “milk” in the product’s name and many are found in the refrigerated aisle next to dairy milk, potentially misleading consumers to believe that these alternative products are of equal nutritional value to milk. Nutrient profile, accessed on webpages of manufacturers of different brands of plant based non-dairy beverages available on the shelves of Canadian supermarkets was compared to that of whole cow’s milk from the Canada Nutrient File database [183]. All products analyzed were original (unflavored) and unsweetened varieties. It is clear from Table 5 that dairy milk is a great source of many nutrients including calcium, vitamins D, as well as protein. Protein quality, which is based on amino acid composition, digestibility, and bioavailability [184], must be taken into consideration as well. Cow milk protein has a >100% DIAAS (Digestible Indispensable Amino Acid Score) which makes it a higher quality protein [184]. Until recently, fortified soy beverage was the second runner up to dairy milk, nutritionally speaking but, as seen from the table, there is a new non-dairy beverage on the block called “non-dairy plant milk” or “pea milk”. It is made from the isolated protein of yellow pea flour. It surpasses dairy milk in protein and calcium content [185], but bioavailability remains unknown. Not all plant-based beverages are made according to the same standards, and fortified versions will have added nutrients in amounts that mimic those found in milk, such as calcium, vitamin D, A, B12, and riboflavin [186]. However, the bioavailability of these nutrients after fortification is not fully known [184]. Vitamin D added to plant-based beverages is in the form of D2 (ergocalciferol) of plant origin and there is scientific evidence pointing to the superior bioavailability of D3 (cholecalciferol) with which dairy milk is fortified [187].

Plant-based dairy substitutes, when consumed as main beverages, can have major health implications especially for young children (1–8 years). A Canadian study reported that consumption of these drinks was associated with lower childhood height [188]. Only cow’s milk and fortified soy beverage are considered nutritious enough for this age group [189]. Protein, calcium, and vitamin D are essential for growth and the latter can be compromised if these nutrients are inadequate in the diet. Furthermore, drinking non-dairy substitutes in children may lead to early satiety, lower hunger, and displace other more nutritious foods. [189]. Many of these substitutes are sweetened with sugar, honey, agave, cane juice, or other sweeteners and contribute empty calories to the diet. The comparison of different nutrients in dairy and dairy substitutes is shown in Table 5.

### 6.3. Exogenous Oral Enzymes

Lactase produced largely from fungi or yeasts can be used prior to or added to dairy meals to aid in digestion of lactose. Enzymes come as gels, liquids, capsules or tablets. A study published about 20 years ago compared three lactase preparations (2–4 capsules or tablets or gels) with 3000 and 6000 IU *β*-galactosidase for milk containing 20 or 50 g lactose. In the case of milk, containing 20 g lactose breath hydrogen, symptoms were significantly reduced. No significant changes were noted with any of the preparations for 50 g lactose loads [190]. In a more recent study two lactase tablets containing 7500 IU were used to test outcome for 25 g lactose in water (lactose equivalent to 500 mL milk). The results were variable in 96 participants all of whom were genetically lactase deficient. About 22% became negative, in about 18% there was a significant reduction in tested breath hydrogen but in the rest there was no significant difference from baseline [191]. Reductions of symptoms did not follow reduction of hydrogen production suggesting symptomatic improvement may have been due to a placebo effect.

Combinations of lactase enzyme with freeze dried yogurt was reported to improve variability of hydrogen and symptoms. In a randomized controlled trial in 24 patients freeze dried yogurt combined with lactase, hydrolysis of lactose was more complete than with lactase alone, however, only 12.5 g of lactose was tried [192]. Additional studies to better refine use of exogenous lactase enzymes could improve outcome. 

### 6.4. Adaptation and Prebiotics for Treatment of Lactose Intolerance

The notion of adaptation to lactose intolerance dates to the mid-20th century when milk powder was provided to some developing countries [193,194]. Initial symptoms of classical LI diminished and often disappeared after about a month of regular milk powder consumption. The physiologic principle of adaptation was demonstrated by Hertzler and Saviano when 16 lactose maldigesting persons with classical breath hydrogen results were converted to those of lactose digesters after 16 days of increasing daily consumption of lactose [195]. There was some symptomatic improvement in gas bloat, but not in diarrhea. As a result, symptomatic improvements may be partly explained as possible placebo impact [69], but the improved breath hydrogen is more difficult to explain. Features of these adaptive characteristics were partly reproduced later, differences explained by different methods of lactose administration [196]. The improvements in symptoms and breath hydrogen response were subsequently also reproduced after regular ingestion of low dose lactulose (galactose, fructose) [197]. Clinical adaptation was also shown to be due to increased numbers of lactic acid bacteria both in vitro [198,199] and in vivo [40,200]. Most recently a clinical trial from China showed that in lactose malabsorbers, four weeks of one cup (250 mL) of whole milk resulted in significant microbial species changes compared with lactose absorbers. However, short chain fatty acid or cardiometabolic markers (mostly serum lipids, glucose, and C reactive protein) did not differ significantly [201].

On a pharmaceutical basis for treatment, regular ingestion of the prebiotic galacto-oligo-saccharide has been shown to improve both symptoms and breath hydrogen response [202]. This product has now been approved by the FDA and is available.

### 6.5. Probiotics

Bacteria which possess β-d-galactosidase are potentially useful for digestion of excess intestinal lactose and thus avoid classical symptoms of LI. Many of the bacteria are lactic acid producers and qualify as probiotics. Probiotics are defined as live microorganisms, when administered in adequate amounts, which confer a health benefit on the host [203]. Probiotics can be administered naturally as part of the fermentation of milk and dairy products as yogurt, kefir, leben, and others. Probiotics can be additionally added to these or administered independently of other foods.

The preferred consumption of yogurt by lactose intolerant people has been observed in many parts of the world. The unique properties of yogurt have been reviewed by Saviano [204]. Yogurt is produced through fermentation by lactic acid-producing bacteria. The traditional organisms are *L. bulgaricus* and Strep thermophiles usually in quantities of 10^8^ colony forming units/mL. Other bacteria with probiotic properties, such as other strains of lactobacilli and bifidobacteria, can be added to produce probiotic yogurt. These bacteria possess *β*-galactosidase (bacterial lactase) which hydrolyses lactose and decreases the pH of yogurt. In addition, it has been found that bacterial digestion of lactose continues in the small bowel. This results in less lactose induced osmotic forces leading to a prolonged orocecal transit time, as well as less gas and abdominal pain [204]. A high lactose content in yogurt is still not suitable for lactose intolerant people [181].

The benefit of consuming probiotics directly is less clear with studies showing variable results. Although probiotics for lactose digestion generally possess *β*-galactosidase, a study by He et al. compared β-galactosidase containing colonic microbiota between lactose tolerant and LI persons and found no statistically significant differences [205]. As noted in the case of yogurt, bacterial lactase is active in the small intestine partly due also to slowing of intestinal transit. There is less information on the impact of probiotic organisms on the general small bowel flora. In a mouse model the administration of *Lactobacillus salivarius* was found to have subtle alternating effects. These were attributed to bacteriotoxic protein production by *L salivarius* [206]. Thus, in the case of direct probiotic consumption, the method of administration and, importantly, the length of administration is less clear.

For example, short-term benefits were shown for administering *L. reuteri* by reduced breath hydrogen and improved symptoms score after 10 days [207]. While another study failed to show any improvement in breath hydrogen or symptoms after 43 days of high dose probiotic VSL#3 (eight lactic acid-producing probiotics) [208]. Still another study using two probiotics, *L. casei* Shirota and *Bifidobacterium breve* showed a benefit in the short-term, after four weeks, and even after three months when the probiotics were already stopped [209].

An earlier systematic review of 10 articles found variable outcomes of either improved breath hydrogen or symptom scores with different probiotics [210]. A recent systematic review however of 15 randomized controlled trials again with 8 different probiotics led the authors to conclude that, while efficacy varied, there was an overall positive benefit to this type of treatment [211].

## 7. Other Treatments Which Encompass a More General Food Intolerance Symptoms

When patients present in a clinic complaining of symptoms lactose does come up in the differential diagnosis, but its role is unclear even after appropriate tests for maldigestion of lactose. Most patients have heard of lactose intolerance and often they have restricted their intake without clear benefit. These patients may have other food intolerances or react to nutrients different from lactose in dairy.

As outlined above symptoms of lactose intolerance can merge with several other causes. Milk intolerance due to fats, A1 casein, or actual milk protein allergy, which is not IgE mediated, can overlap. In children Cow’s Milk Protein allergy is more significant, while in adults this condition is rare and may overlap more with symptoms of LI. The role of genetically-determined casein in cows causing symptoms requires further evaluation. In others several diseases, particularly celiac and Crohn’s disease, are conditions to consider in younger patients, while several other diseases, like microscopic colitis and others, need to be considered in older populations.

Comparison of treatments for LI and CMPA is outlined by Heine et al. The authors emphasize that CMPA is not mediated by lactose and is a type of allergic reaction either to IgE with potential anaphylactic reaction of non-IgE, which can manifest as an enteropathy, sometimes even mimicking colitis of inflammatory bowel diseases. Heine et al. point out that lactose restriction may only be necessary with CMPA induced enteropathy which can give rise to secondary lactose maldigestion and LI. Otherwise special formulae are used and these may also have lactose [180]. Lactose in these situations added to extensively hydrolyzed formulas can enhance calcium absorption. In young infants addition of lactose can favorably alter the colonic microflora [40].

When symptoms persist (diarrhea, abdominal pain, alterations in bowel movements with or without bloating, and the diseases have been reasonably ruled out), irritable bowel syndrome or other food intolerances need to be considered, although pharmaceutical treatments or other probiotics may be available several diets have been introduced that may help.

Of these the FODMAP diet (fermentable oligo di, monosaccharides, and polyols) is one of the most popular [212]. This is defined as a “short-term (2–6 week) restriction of foods high in fermentable carbohydrates, followed by re-challenges to assess tolerance” [213]. The rationale for the diet is based on similar concepts as for LI (described by He et al. [6]). Different carbohydrates reaching the colon cause osmotic build up and formations of short chain fatty acids. There have been successes reported with this diet [214]. In one study of 473 patient who responded to the FODMAP diet, both fructose and lactose malabsorption with hydrogen or methane production predicted adequate response [215]. Since many foods are involved dietary guidance is necessary to reintroduce different food items.

The other popular course is a gluten free diet (GFD) for presumed gluten sensitivity but without diagnostic features of celiac disease. The presence of the genetic markers necessary for celiac disease (HLA DQ-2 and 8), may increase the chance of clinical response in the opinion of some authors [216]. Others feel that GFD is also a marker for a low FODMAP diet [217]. A recent study by Skodje et al. seems to support this notion by reporting that fructans rather than gluten is responsible for improving symptoms [218].

Recent systematic reviews suggested that due to a number of biases, the outcomes with FODMAP may be more in keeping with a placebo response [219]. Another systematic review found that based on two randomized controlled trials of 111 patients with IBS, GFD did improve global symptoms, albeit non-significantly [219]. The same publication reported seven randomized controlled trials with 397 patients and again found that FODMAP did improve global symptoms and significantly. However, the quality of data was deemed very low [220].

Where these diets will ultimately fit into treatment of gastrointestinal symptoms with dairy, therefore, is not yet clear, but do serve as other possible therapeutic options.

## 8. Summary

The term lactose intolerance currently encompasses a more complex meaning than in the past. The continued concept that lactose maldigestion is equivalent to lactose intolerance confuses understanding of the proper place of lactose specific management in lay persons’ and perhaps even the scientific community’s perceptions. Outcomes of lactose challenge tests with resulting symptoms do not accurately reflect every day consumption of lactose, when combined with other foods. The symptom complexities of lactose intolerance could be modified by other food intolerances and the presence of functional gastrointestinal disorders. Although the most common disorder of irritable bowel syndrome is separate from LI, the presence of LM in IBS aggravates symptoms after lactose consumption. However, LP persons can also react to both lactose challenge as well as dairy food consumption. In these cases, research on non-lactose related causes of symptoms is important for the future. 

These perceptions may lead to removal of dairy foods from diet. Although, research into substitutes are advancing to replace dairy this substitution may not be necessary and may have some disadvantages. In western societies dairy remains the main source of calcium and other nutrients for bone development. As outlined the advantages for dairy seem to favor health outcomes for the most part. Dairy foods are not likely the primary cause of a few diseases that have shown some increased risks. Current research favors beneficial impact on a number of different pathogenic processes. In this paradigm specific nutrients and matrices have an impact on the host and the microbiome. The impact of dairy on diseases requires continued evaluations as new methods of study, such as Mendelian randomization are applied. 

## Figures and Tables

**Table 1:** Classification of four causes of lactose maldigestion. References are found in the appropriate sections of the text.

Congenital Lactase deficiency [34,35,36,37]	Rare mutation at the lactase gene site described initially in Finland, now a few cases elsewhere also. It is associated with severe diarrhea acidosis and hypercalcemia.
Developmental Lactase deficiency [39,40]	Premature neonates usually have insufficient intestinal lactase Some term neonates may also have incomplete development of the enzyme
Primary Adult Lactase deficiency [42]	The most common cause of lactase deficiency. This is due to several polymorphisms in the transcription promoter region of the lactase gene. The ability to digest lactose into adulthood is a dominant trait and affects about 1/4–1/3 of the world’s population.
Secondary Lactase deficiency [44]	Diseases or toxins which affect the proximal small intestine can lead to loss of intestinal surface area with resulting lactase deficiency. After recovery the surface can improve, and unless there is also genetic predisposition lactose digestion may improve. Some examples of diseases are viral illness, e.g., Rota virus infection in children, unicellular parasites, e.g., Giardia, celiac disease, malnutrition, radiation exposure, upper gastrointestinal surgery, and some medications, e.g., olmesartan.

**Table 2:** Terms used to describe lactose digestion status.

Term	Definition
Lactase Persistence (LP)	A dominant genetic trait usually associated with continued high levels of lactase production into adulthood.
Lactase Non-Persistence (LNP)	A recessive and ancestral genetic trait associated with a decline in intestinal lactase to < 10 u/g of tissue sometime between the end of weaning and adulthood.
Lactase Deficiency (LD)	Reduction of intestinal lactase enzyme from any cause, either genetic (LNP) or any secondary causes, like diseases or injury of the proximal small bowel mucosa.
Lactose Maldigestion (LM)	Inability to digest lactose for any reason, primary LNP, but also secondary causes. Most common tests for lactase deficiency are actually for LM.
Lactose Intolerance (LI)	Adverse symptoms resulting from the ingestion of lactose, including flatus, gas, bloating, cramps, diarrhea and, rarely, vomiting. LI may occur without LM.
Self-Reported LI (SRLI)	Persons believing themselves to be lactose intolerant without testing for LM. Nocebo and psychological characteristics may play a role in milk avoidance.
Lactose Sensitivity (LS)	Adverse symptoms with or without symptoms of LI and may also include depression, headache, and fatigue, with or without LM. LS symptoms may overlap with Irritable Bowel Syndrome.

**Table 3:** Observational studies and meta-analyses show possible effects of different dairy products on 19 conditions. In the case of bone calcium from dairy products is the most consistent in western societies. However, bone osteoporosis and fractures are controversial.

Disease	TDF	Milk	Lfat	Hfat	Lactose	Cheese	Yogurt	Ferment	Calcium
**Bone**	**-**	**-**	**-**	**-**	**? -**	**-**	**-**	**-**	**-**
**MetS**	**-**								
**CAD**	***n***	***n***	***n***	***n***		***n***	***n***	**-**	**? -**
**CVD**	***n***					***n***	***n***		
HTN *	***-***	***n***	***-***	***n***		***-***	***n***	**-**	
**Stroke**	**-**	**-**	**-**						
T2Dm *	**-**	***n***	***-***	***n***		**-**	**-**	**? *n*/+**	
Obes *	**?-/? *n***	**?-/?*n***							
**CRC**	**-**	**-**					**? -**		**? -**
**IBD**	**-**	**-**							**-**
**Brst ca**	**? - **		**-**	**? -**			**-**		**-**
**Ovar ca**	**? +**	***n***	***n***		**? *n***		**? *n***		**? -**
**Sto ca**	**? -**	***n***							
**Pros ca**	**+**		**+**			**+**			**+**
**Test ca**		**? +**							
**Lun ca**	***n***								
**Panc ca**	***n***								
**Blad ca**	**? -**								
**NHL**	**? +**	**+**				**+**	***n***		

* It is noted that the outcomes of Mendelian randomization studies which included the genetic polymorphism as an instrumental variable did not show that dairy intake reduced obesity, hypertension, or type 2 diabetes [125,126,130,132]. However, a study evaluating risk of milk in cardiovascular disease did not find any increased risk [127]. The sign - denotes reduced risk, *n*—neutral effect and + denotes increased risk. The ? indicates varied outcomes reported. The / symbol represents divergent reports. TDF—total dairy foods, Lfat—low fat milk, Hfat—high fat milk, Ferment—refers to fermented dairy. MetS—metabolic syndrome, CAD—coronary artery disease, CVD—cardiovascular disease, HTN—hypertension, T2D—type 2 diabetes, CRC—refers to colorectal cancer, IBD—inflammatory bowel disease, Brst ca—breast cancer, Ovar ca—ovarian cancer, Sto ca = stomach cancer, Pros ca—prostate ca Ovar ca—ovarian cancer, Test ca—testicular cancer. Lun ca—lung cancer Panc ca—pancreatic cancer, Blad ca—bladder cancer, NHL— non-Hodgkin’s lymphoma. The table is based on references [99,100,116,117,118,133,134,135,147,148,149,153,154,155,156,157,161,163,164,165,166,167,168,170,173,174,177,178,179]. Relationships between specific diseases and references are noted in the text.

**Table 4:** Recommendations for management of diagnosed lactose intolerance.

1. Gradual introduction of cow milk	Start with 30–60 mL per day and gradually increase to a maximum of 250 mL per day. Consume with meals rather than on an empty stomach to slow release of lactose in small intestine [181]. Higher fat milk may be better tolerated due to slower transit time. Consistency of consumption on a daily basis is key to building tolerance
2. Inclusion of aged cheeses	Generally well tolerated due to their low lactose content (0.1–0.9 g of lactose in 30 g of hard cheese) [180]
3. Inclusion of lactose reduced milk products	These are nutritionally identical to regular milk products [181]
4. Use of lactose tablets and drops	Can be taken prior to consuming dairy foods or simultaneously with dairy meal
5. Inclusion of other food sources of calcium such as dark green leafy vegetables, dried beans and legumes	This can help boost the intake of this mineral. Green leafy vegetables have the added benefit of contributing Vitamin K which plays an important role in calcium regulation and bone formation. Calcium bioavailability from these foods is lower than that from dairy due to the fibers, phytic, and oxalic acids [182]. Amounts of non-dairy foods evaluated to provide same amount of calcium from one serving of dairy (250 mL milk) are as follows: 1.1 servings fortified soy beverage 1.2 servings of bony fish 2.2 servings of green leafy vegetables It is important to note that these foods do not provide the equivalent profile of other nutrients and amounts needed can be unrealistic to consume in some cases [180]
6. Consumption of fermented products like yogurt	These are produced by bacterial fermentation of milk lactose into lactic acid. Yogurts are also a source of probiotics and prebiotics, and both exert beneficial effects on gastrointestinal microflora [181]. Cultured bio yogurts and cultured milk blends which contain additional bacterial strains have become more available in recent years. However, results from an Israeli survey of leading brands showed a high lactose content not suitable for lactose intolerant people [181]. The rationale on the use of yogurt is further discussed below in conjunction with probiotics

**Table 5:** Nutrient profile comparison of whole cow’s milk with non-dairy beverages.

NUTRIENT CONTENT per 250 mL	unit	Cow’s Milk Whole *	Soy Beverage **	Plant Milk Beverage **	Almond Beverage **	Coconut Beverage **	Cashew Beverage **	0at Beverage **	Rice Beverage **	Hemp Beverage **
energy	k/cal	157	90	110	30	50	25	130	130	60
protein	g	8	8	10	1	0.2	1	4	1	3
Total fat	g	8	4.5	5	2.5	4.5	2	2	2	4.5
carbohydrate	g	12	4	6	1	1	1	25	27	0
calcium	mg	291	300	450	300	300	300	121	360	282
sodium	mg	111	90	140	160	35	160	105	N/A	110
potassium	mg	340	360	330	35	30	N/A	133	N/A	100
zinc	mg	0.95	1.1	1.6	1.1	1.1	1.1	N/A	N/A	1
iron	mg	0.08	1.44	N/A	0.36	0.36	0.72	1	1	2
Vitamin A	IU	396	300	366	300	300	300	N/A	300	N/A
Vitamin D	IU	104	270	160	270	270	270	60	150	6µ0
Vitamin B12	µg	1.16	1.2	2.7	1.2	1.2	1.2	N/A	N/A	N/A
riboflavin	mg	0.4	0.325	N/A	0.325	0.325	0.325	N/A	N/A	N/A

* skim, 1%, 2%, and whole milk only differ in amount of calories and fat. ** for select brands. Not representative of all brands. N/A—not available.
